# Contemporary perspectives and debates in student ultrasound education: exploring didactic elements and theoretical aspects

**DOI:** 10.1186/s12909-026-09089-8

**Published:** 2026-06-19

**Authors:** F Recker, SL Sänger, Y Dong, A Gschmack, C Jenssen, K Möller, R Neubauer, M Blaivas, MI Prats, J Ruppert, R Sirli, J Weimer, SC Westerway, C Zervides, CF Dietrich

**Affiliations:** 1 University Hospital Bonn Venusberg Campus 1, Bonn, 53127 Germany; 2 Medizinische Fakultät Carl Gustav Carus, Technische Universität Dresden, Dresden, Germany; 3 Department of Ultrasound, Xinhua Hospital, Shanghai Jiao Tong University School of Medicine, Shanghai, China; 4 Justus-Liebig-Universität, Gießen, Germany; 5 Dept. for Internal Medicine, Krankenhaus Märkisch Oderland, Strausberg, Germany; 6 Brandenburg Institute for Clinical Ultrasound (BICUS) at Brandenburg Medical University, Neuruppin, Germany; 7 Medical Department I/Gastroenterology, SANA Hospital Lichtenberg, Berlin, Germany; 8 Department of Medicine, University of South Carolina School of Medicine, Columbia, South Carolina USA; 9 Department of Emergency Medicine, The Ohio State University Wexner Medical Center, Columbus, Ohio USA; 10 Department of Medicine, Justus Liebig University Giessen, Giessen, Germany; 11 Center for Advanced Research in Gastroenterology and Hepatology, Department of Gastroenterology and Hepatology, ”Victor Babeș” University of Medicine and Pharmacy, Timișoara, Romania; 12 Rudolf Frey Learning Clinic, University Medical Center, Johannes Gutenberg University Mainz, Mainz, 55131 Germany; 13 School of Dentistry & Medical Sciences, Charles Sturt University, Wagga Wagga, Australia; 14 CZMH Medical Physics and Dosimetry Services LTD., Limassol, Cyprus; 15 University Hospital Frankfurt, Johann-Wolfgang-Goethe University, Frankfurt/Main, Theodor-Stern-Kai, Frankfurt am Main, 70596 Germany

**Keywords:** Ultrasound Medical Education, Training, Ultrasound, Education, Didactic elements

## Abstract

**Background:**

Medical ultrasound education is evolving, embracing various teaching methods such as classical, e-learning, and hands-on approaches. The integration of ultrasound into medical school curricula has highlighted the importance of blended learning, although there is limited literature on specific learning theories and pedagogical concepts. Medical ultrasound learning is unique, requiring psychomotor and technical skills in probe handling, anatomical and clinical knowledge, and cognitive abilities for image interpretation.

**Methods:**

A review of literature in a systematic way was conducted across multiple databases, including PubMed, Embase, and Scopus, using predefined search terms such as “ultrasound education,” “e-learning,” “simulation-based ultrasound,” and “peer-assisted learning ultrasound.” The search targeted studies focusing on undergraduate medical education and ultrasound instruction. Following duplicate removal, two independent reviewers screened titles and abstracts, with eligible full-text articles assessed against inclusion criteria. Studies were included if they addressed didactic ultrasound teaching methods and reported on educational outcomes relevant to medical students or trainees.

**Results:**

The inverted classroom approach, where preparatory material is studied before class, was effective in ultrasound education. Blended learning, an educational approach that combines traditional classroom instruction with online learning activities and resources, enhanced both cognitive understanding and practical skills. Simulation-based training emerged as valuable, providing safe environments for learning and is applicable across pre-clinical and clinical phases. The study also assessed the advantages and limitations of simulation-based training and e-learning.

**Discussion:**

The paper highlights the need for diverse teaching methodologies in ultrasound education. It emphasizes that while traditional methods may be cost-effective, modern approaches, such as blended learning and simulation-based training, offer more engaging, practical, and efficient learning experiences. Integrating these methods within existing curricula enhances ultrasound training quality, advocating for an interdisciplinary and technologically adapted approach.

**Conclusion:**

The study concludes that a blend of traditional and contemporary teaching methods, including e-learning and simulation, is essential for effective ultrasound education in medical studies. Adapting to technological advancements and diverse learning styles is crucial in preparing students for modern healthcare demands.

**Supplementary Information:**

The online version contains supplementary material available at 10.1186/s12909-026-09089-8.

## Introduction

The increasing importance of ultrasound as a diagnostic tool across many fields of medicine has resulted in its further integration into medical school curricula [1–3]. When teaching and implementing ultrasound training programs, medical educators should consider several aspects. Learning ultrasound differs from acquiring other practical clinical skills, where a straightforward procedure can be practiced with an identical outcome. Ultrasound, however, requires psychomotor skills in manipulating the ultrasound probe, technical skills to understand machine settings, anatomical and clinical knowledge, and cognitive capabilities in searching for diagnostic information in the ultrasound image. Ultrasound examinations can be challenging to standardize as diagnostic procedures, because their outcomes may vary depending on operator skill, patient anatomy, and clinical context, making them less predictable than other imaging modalities [4]. The learning process in ultrasound training can be divided into different steps, which can run consecutively or simultaneously. In general, components of ultrasound education can be differentiated into theoretical training (lectures, seminars, e-learning, etc.) and practical training (in small groups or one-on-one, using either student models or patients, or with simulators). An initial teaching of the theoretical basics creates the foundation for subsequent hands-on practice. Technical knowledge is required in order to be able to handle the ultrasound device correctly and the awareness of ultrasound physics enables an understanding of image production and artifacts. Furthermore, a recall of anatomy helps the students to better orientate themselves during the ultrasound scan. Students should be aware of indications and limitations, as well as safety issues of ultrasound and ultrasound-guided procedures. Learning to properly document ultrasound examinations is also crucial for later clinical practice. In summary, the knowledge taught during the theoretical lessons in ultrasound education is essential for the development of translational skills such as image interpretation or medical decision making [5, 6].

When looking at the conduction of knowledge transfer in student ultrasound education (SUSE), various factors play a role in the implementation and evaluation of didactic approaches and educational materials. With the ultimate goal of providing students with the best possible education, assessment of the efficiency of the didactic method plays a major role. This is usually achieved by the assessment of learning outcomes of students. The effectiveness of training methods depends on various individual, social and structural factors. For example, different learning types and existing relevant prior knowledge, such as anatomy or physical examination, can influence the personal response to a didactic method [7, 8]. In the past, it has also been investigated whether gender has an influence on the preference of a didactic method [8, 9]. In addition to personal aspects, the effectiveness of a teaching approach is particularly determined by its implementation. Different approaches may require different levels of organizational effort and personnel commitment, therefore may be less or more suitable for the respective institution. Didactic concepts include conventional lectures, self-directed learning, inverted and flipped classroom formats, blended learning, problem-based and team-based learning, peer teaching, as well as the combination of different approaches and the integration of simulation and gamification tools.

Several publications investigated the implementation of different approaches. For example, Cartier et al. used a learning theory approach to design an ultrasound course for medical students with cognitive, behavioral, and constructivist learning components [4]. The European Federation of Societies for Ultrasound in Medicine and Biology (EFSUMB) recommends a constructivist approach to teaching during pre-clinical training, where knowledge is taught through organ and topic-specific modules incorporating clinical aspects [1].

The current landscape of didactic ultrasound training is marked by ongoing debates and challenges, particularly concerning theoretical components such as blended learning, simulation-based training, the concept of an inverted classroom, traditional lectures, and e-learning. Thus the research questions were:

- What are the strengths, limitations, and learning outcomes of different didactic teaching strategies (e.g., traditional lectures, e-learning, flipped/inverted classrooms, blended learning, simulation-based training, peer-assisted learning, gamification) in undergraduate medical ultrasound education?
- How can these diverse teaching strategies be optimally integrated into the undergraduate medical curriculum to enhance students’ ultrasound learning outcomes?

In this paper, we summarize the current situation of didactic teaching elements, including its existing controversies, and derive the resulting conclusions for ultrasound training in medical studies.

## Methods

We conducted a review of literature in a systematic way to gather current evidence on didactic methods in undergraduate ultrasound education. PubMed and Google Scholar were searched from inception through March 2024 using combinations of terms such as “ultrasound education,” “medical student ultrasound training,” “didactic ultrasound,” “blended learning ultrasound,” “simulation ultrasound training,” “e-learning ultrasound medical education,” “inverted classroom ultrasound,” and “peer-assisted learning ultrasound.”

**Fig. 1:**
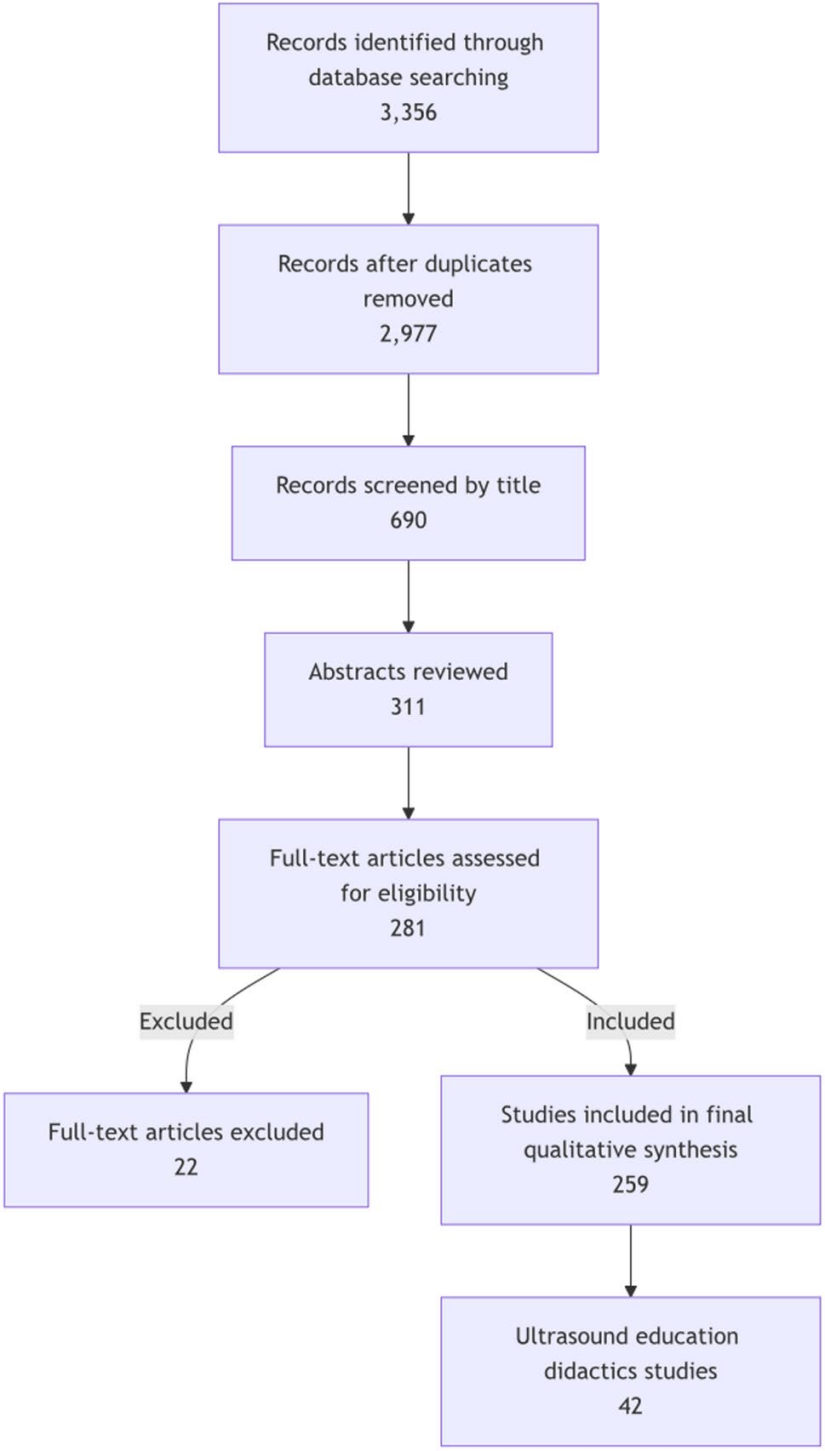
Flow-Chart of the included studies in this review according to PRISMA-Style guideline

A review of literature in a systematic way was conducted using PubMed and Google Scholar databases to identify studies on undergraduate ultrasound education published up to March 2024 (Fig. 1). The search strategy included combinations of the following keywords: “ultrasound,” “undergraduate,” “didactics,” “teaching,” “curriculum,” “medical student,” and “education.” Duplicate records (*n* = 379) were removed automatically before screening. Two reviewers (F.R. and R.N.) independently screened the titles (*n* = 690) and abstracts (*n* = 311), resolving disagreements through discussion. Full-text articles were retrieved for 295 records, of which 281 were assessed for eligibility. After applying inclusion and exclusion criteria, 259 articles were included in the final review, comprising 42 original ultrasound didactics studies. The selection process adhered to the PRISMA 2020 guidelines.

## Results

We organized the results first by *learning modes* and then by *pedagogical strategies*.

### Learning modes

#### Traditional lecturing

A frequently used approach is the transfer of knowledge within traditional lectures. A major advantage of this method is the high number of students that can be educated simultaneously. However, during large plenary sessions, little attention can be paid to differing individual learning styles or levels of prior knowledge. The impersonal atmosphere can make it difficult for students to ask questions or request revision. In the digital age, conducting face to face lectures reduces flexibility and accessibility of medical education and can lead to a reduced number of attendees. Traditional lectures often focus on a unilateral knowledge transfer, with students taking a more passive role, which decreases attention and can reduce their motivation. While both traditional and online lectures allow for standardized delivery of content, important differences exist between the two formats. In traditional face-to-face lectures, all students are exposed simultaneously to the same material, which enables comparable levels of knowledge to be assumed at the end of the course. Online lectures, particularly when recorded, can be even more standardized, as every student receives the identical version of the content. However, traditional lectures typically provide greater immediacy of interaction, spontaneous feedback, and nonverbal communication between instructors and students, which may enhance engagement and clarify misunderstandings in real time. In contrast, online formats offer flexibility and the opportunity for asynchronous repetition of content, but they are more susceptible to reduced attention and disengagement unless interactive features are deliberately integrated. Moreover, online teaching requires adequate digital infrastructure and competencies, while traditional lectures depend primarily on physical presence and scheduling. Thus, while both formats support standardization, they differ substantially in terms of interactivity, flexibility, and the conditions under which student engagement can be achieved [9]. The organizational effort is low, as one lecturer can train a large number of students and prepared presentations can be used several times. In the modern era, video-recorded lectures combine some of the advantages of didactic lecture with asynchronous online learning. Students can then learn in their own time, possibly adjusting the speed of the content to fit their needs. The consequence to this method compared to traditional in-person learning is less potential for interactivity with peers or the teacher. In the field of ultrasound training, this approach is particularly beneficial for teaching cognitive components of ultrasound. These include the underlying physical principles of ultrasound, indications and limitations of certain ultrasound examinations, and strategies for image interpretation.

#### Self-directed learning

Self-directed learning transfers most of the responsibility for theoretical education on the students themselves, ensuring they take the initiative for their own learning progress. Learning outcomes are strongly contingent upon the individual student’s intrinsic motivation and persistence [10]. In return, the organizational effort on the part of the institution is minimal. By handing out learning materials such as scripts, text books, or links to relevant literature, a certain, but limited degree of control over the scope of learning can be achieved. Institutions should consider personal attributes of the learners such as internal and external factors that motivate them and should provide a supportive environment [11]. Both analysis, synthesis and application of acquired knowledge should be part of the learning process. Educational pedagogy should not be limited to the bare knowledge transfer but also include an honest self-reflection to identify existing gaps in knowledge and assessment to ensure sufficient competence [11].

Self-directed learning allows students to organize their learning material themselves and work flexibly in terms of time and location. Self-directed learning study materials should include high quality images and films, with clear explanations and markers for normal and pathologic findings, so that there is no place for misinterpretation [12]. In this way, students can implement their own personal learning preferences. Thereby, self-directed learning can strengthen personal self-organization and help students to reflect on knowledge gaps [13]. However, self-directed learning lacks any form of social interaction and students are not given the opportunity to profit from problems and experiences of fellow students. In general, self-directed learning of medical advances should be a life-long process for every physician [11]. Therefore, an early practice and acquisition of personal learning strategies can be very beneficial in the long term.

#### Inverted classroom

The core concept of an inverted classroom is that students encounter information before class, thereby using class time for activities that involve higher order thinking. In the case of ultrasound modules, providing teaching materials before the course can facilitate the preparation of the participants for the ultrasound course. Heinzow et al. found that encouraging students to make schematic drawings of ultrasound images helped to strengthen their understanding of anatomy, especially concerning cross sections in the abdomen [5]. By initial independent study of specific literature, different levels of prior knowledge can be assimilated more easily [14]. Shifting parts of the theoretical course content to independent learning facilitates lower-order cognitive processes, including the acquisition of knowledge and the understanding of conceptual relationships. In-class sessions may focus on subsequent stages of learning and higher-order learning activities, such as the analysis, application, and evaluation of knowledge, as well as practical exercises [14]. Furthermore, more time for practical exercises, such as hands-on training, can be offered, which leads to higher motivation, better long-term recognition and greater persistence of knowledge [15]. In addition to this, more time can be devoted to questions and ambiguities. A potential disadvantage of the inverted classroom concept is that it relies on students taking responsibility for their own learning progress. If students attend courses unprepared it could lead to frustration of well-prepared students due to an unplanned theoretical briefing and resulting delay. Finally, the inverted classroom concept combines the advantages of self-directed learning with the supporting presence of ultrasound experts [15–17].

#### E-learning & blended learning

In the rapidly evolving field of ultrasound medical education, the prominence of online or electronic learning (e-learning) has been significantly accentuated, especially in the wake of the COVID-19 pandemic [18, 19]. This form of education encompasses an extensive array of digital resources, including electronic textbooks, educational websites, instructional videos, online modules or courses, webinars, podcasts, and an active presence on various social media platforms. The content available in this digital spectrum ranges from commercially produced materials, akin to traditional textbooks and in-person courses, to a growing collection of freely accessible resources known as Free Open Access Medical Education (FOAMed) [20].The advancements in electronic education platforms, coupled with the dynamic evolution of social media, have fundamentally altered the educational landscape, aligning with the current generation’s tendencies towards more personalized, autonomous learning approaches [21–23].

The advantages of e-learning in ultrasound education are multifaceted. One of the primary benefits is the unprecedented level of accessibility it provides; learners can access educational content from virtually any location with an internet connection [24–26]. This accessibility significantly enhances the flexibility with which learners can engage with the material, allowing them to adjust the pace of learning to suit their individual needs. Unlike traditional classroom settings, e-learning enables students to control their learning environment, pacing, and content repetition, thereby facilitating a more personalized learning experience. Moreover, the integration of e-learning into ultrasound medical education offers unique opportunities for exploring and understanding rare cases, often not encountered in typical clinical environments [27–37].

Despite these strengths, e-learning is not without its challenges. The primary concern lies in the quality and reliability of the content available. The vast expanse of online resources comes with the risk of encountering misinformation, as the credentials of content creators may not always be verifiable [38]. Additionally, many e-learning resources may not undergo the rigorous peer-review process typically associated with published journal articles or textbook Chaps. [39]. Consequently, there is a necessity for careful scrutiny and validation of these resources, which can be achieved through developed metrics aimed at evaluating the quality of free online educational materials [40, 41]. In this regard, professional societies, who are already involved in establishing standards for various domains of ultrasound, should be implicated in developing e-learning tools dedicated to students’ education.

Another significant challenge is the potential lack of comprehensive coverage in the ultrasound curriculum through e-learning alone. The goal of many free online resources is to attract the widest audience possible, which can sometimes lead to a bias towards more engaging or topical content at the expense of comprehensive educational coverage [42]. This issue is particularly pertinent for learners who rely solely on independent online learning, separate from a structured educational program.

In response to these challenges, the concept of blended learning has emerged as a potent solution [43–45]. Blended learning effectively combines the benefits of e-learning with the indispensable aspects of traditional in-person hands-on training. This approach not only enhances knowledge outcomes but also ensures a more rounded educational experience by integrating e-learning resources both before and after practical training sessions [46]. The flexibility afforded by e-learning allows for its effective distribution, facilitating periodic review and improved retention of knowledge [47, 48]. The future of ultrasound education appears to be heading towards an increased adoption of advanced simulation, virtual reality, and artificial intelligence, offering even broader opportunities for remote learning [26, 29, 48–50].

In conclusion, an efficient approach in ultrasound medical education could be encompassed by an harmonious blend of e-learning and traditional training methods. This blended learning model leverages the strengths of digital accessibility and flexibility while ensuring the crucial hands-on experience and mentorship that only in-person training can provide [51]. As ultrasound education continues to evolve, it becomes imperative for educators to embrace and integrate these diverse modalities, crafting curricula that not only engage learners but also equips them with comprehensive, practical skills essential for their future roles in healthcare (Table 1).

**Table 1:** Potential advantages and limitations of different didactic approaches. For more information using M-learning see [34, 52]. It provides a comparative summary of didactic approaches discussed in this review

Teaching methods/ Evaluation parameters	Self-directed learning	Lectures	Peer-assisted learning	Team- / problem- based learning	Case presentations	E- Learning	Blended learning	Inverted classroom	Gamification
Quantitative content transfer	+++	+++	++	++	+++	+++	+++	+++	+
Standardization of content transfer	-	++	+	+	+	+++	++	++	-
Control of final learning outcome	-	+	+	+	+	++	++	++	-
Interactivity	-	-	+	+++	++	++	+	+	+++
Organizational effort for educators	-	+	+	++	-	+	++	+	+++
Flexibility, Accessibility	+++	+	+	+	+	+++	++	++	-
Social interaction	-	+	+++	+++	+	-	+	+	++
Learning atmosphere	+	+	+++	++	+/-	++	+	+	+/-

Note: The table reflects the authors’ qualitative synthesis of the literature combined with our collective teaching experience. The symbols (+++, ++, +, –) represent our subjective assessment of each method’s relative advantages or limitations. For example, “+++” indicates a strong advantage or strength, “++” a moderate benefit, “+” a slight benefit, and “–” a notable limitation. These ratings are not absolute; in practice, each method’s effectiveness can vary depending on the educational context, resources, and learner group. The table is intended to guide general comparison rather than serve as a precise metric

### Instructional strategies

#### Case-based presentations and discussions

Case presentations by students can increase the active participation of students in the course. Changing lecturers / presenters often makes the course more diverse and interesting. In addition to the actual content of case studies, students also practice evaluating scientific literature, giving presentations and explaining complex content in a more comprehensible way [53–55].

#### Peer-assisted learning (PAL)

Peer teachers are frequently used in ultrasound training in order to establish the lowest possible teacher-student ratio. This can result in a more conducive learning atmosphere that encourages students to ask questions and allows repetition of skill practice. Concepts such as social and cognitive congruence attribute peer teachers with an increased understanding of the didactic preferences of their fellow students. This enables them to better empathize with, and potentially design their teaching closer to the students’ needs, as well as explaining content more comprehensibly [56–59]. However, in a study by Li et al. it was shown that theoretical content was taught more effectively by lecturers rather than by student tutors. As experts, lecturers may have a greater awareness of the need for a theoretical foundation before moving on to practical skills [60]. The effectiveness of peer-teachers in ultrasound education seems to be dependent on the previously existing anatomy knowledge of the trainees [7]. Nevertheless, several studies that examined peer teaching in undergraduate ultrasound education state peer teaching to be comparable or even superior to conventional teaching by professional lecturers [61]. In addition, studies have demonstrated the long-term effectiveness of peer teaching [62, 63].

#### Team-based and problem-based learning

While students predominantly take on a passive role in traditional lectures, team-based and problem-based learning requires the active participation of the learners. Through interactive exchange and reinforcement, students may learn from each other through applying different individual approaches and ways of thinking. Team-based learning promotes interdisciplinary cooperation and constructive joint decision-making. In addition, an inter-individual constructive exchange and discussion is encouraged [64]. Building the course around a clinical case in the context of problem-based learning can promote the clinical relevance of the theoretical unit and increase the students´ motivation. In addition to ultrasound-specific learning content, the use of problem-based learning simulates the search for an appropriate diagnosis for particular symptoms and physical conditions. The disadvantages, however, are a higher degree of organizational effort to design the case studies and limited control over learning outcome, as the experiences of the students in the individual groups can differ. A study by Cremerius et al. showed that team-based learning is even more effective than the training by peer tutors [8, 65, 66].

#### Gamification

Gamification is an approach that uses elements of scoring systems or incentives to create a challenge that can be mastered alone or through teamwork. Often chosen as a final additional module that also has an assessment purpose, students can apply what they have previously learned in a clinical context. The approach encourages students to actively recall their knowledge in a stressful situation and thus supports the sustainable retention of knowledge. Examples of gamification of ultrasound training can be found in the Ultrasound Challenge by Bahner et al. or the Sono Slam by Boulger et al. [67, 68]. Supporters of the approach hope that gamification will increase students’ motivation to learn further clinical skills despite an overloaded curriculum. In addition, Hennekes et al. were able to show that gamification promotes students’ self-confidence their autonomy and ability to work in a team [69].

It should be noticed, that the gamification approach to learning is not equally suitable for all personality types and maybe perceived as unpleasant and stressful by less proactive learners. With regard to the comparability of the learning success of individual students, gamification approaches tend to assess the extent to which students can master the situation or solve the task with the skills they have acquired. The individual self-reflection of the students allows them to better identify existing knowledge gaps at the end of the challenge. Gamification approaches often require greater organizational effort and well-thought-out planning, which makes their implementation more difficult.

#### New teaching methods vs. theoretical training with lectures

The World Federation of Ultrasounds in Medicine and Biology (WFUMB) acknowledges the cost-effectiveness and established nature of traditional teaching methods. These methods utilize existing infrastructures and do not require the retraining of educators or new teaching strategies, facilitating large group instruction [70]. Studies indicate that incorporating ultrasound lectures into the medical curriculum yields beneficial outcomes, providing clarity on ultrasound usage [28, 71–73], enhancing student interest in anatomy and physiology, and improving diagnostic skills [74].

Innovative teaching methods, such as electronic (e-) and mobile (m-) learning [34, 52] and the use of social networks, offer shorter, more effective communication channels [1, 70]. Gamification, as adopted by the Ohio State University College of Medicine in their annual Ultrasound Challenge, adds diversity to traditional curricula, boosting student engagement through competition and the development of skills in high-pressure scenarios [67].

Well-integrated new programs in existing curricula can enhance ultrasound training quality [75–78]. In the pre-clinical phase, the focus should be on reinforcing students’ understanding of anatomy, physiology, and disease pattern recognition [79]. Additionally, a foundational understanding of cross-sectional imaging is crucial for advancing in ultrasound diagnostics. In the clinical phase, the emphasis shifts to the practical application of ultrasound in diagnostic imaging, including teaching the indications and limitations of ultrasound examinations [79]. An interdisciplinary approach in both study phases is vital for effective integration and implementation in medical degree curricula.

University hospitals are encouraged to offer Ph.D. programs in ultrasound, allowing students to contribute to the scientific advancement of its applications. Motivation, a core component of successful learning as per Edward Deci and Richard Ryan’s self-determination theory, should be fostered in students, promoting interest in ultrasound, and facilitating a sense of competence, autonomy, and social integration. This theory’s relevance was underscored during the COVID-19 pandemic, as evidenced by a multi-country study led by Elisabeth Pelikan and colleagues from the University of Vienna’s Institute for Psychology of Development and Education [80]. Effective student learning also reduces tutor training costs, as peer-to-peer teaching becomes increasingly favored in German medical schools. Well-trained students can serve as future tutors, requiring shorter training periods and achieving early independence in their roles [66, 81].

## Discussion

Fundamental knowledge of examination techniques, indications and limitations, as well as an understanding of sono-anatomy and the development of sonomorphologic pathologies and artifacts is essential in order to be able to apply practical skills. In addition, basic understanding of the physics behind the generation of ultrasound images is necessary to be able to correctly interpret acquired images and subsequently make a therapeutic decision.

In an overcrowded curriculum, it is worthwhile to use the most efficient and effective didactic approaches for both learners and lecturers. The investigation of diverse didactic approaches in various ultrasound applications supports an evidence-based selection of the appropriate method. Nevertheless, each didactic method comes with different advantages and limitations. While self-directed learning tends to strengthen students’ own organizational skills and personal responsibility [11], team-based learning for solving problems tends to promote collaborative, constructive teamwork [64]. Despite the reduced possibility of responding to individual questions and needs, traditional lectures offer an opportunity to present teaching content to a large number of students. Besides the personal impact of different approaches on the individual student, the successful implementation of the ultrasound training should also be planned with realistic ideas regarding the organizational effort, the availability of lecturers and financial resources. While there are not any recommendations preferring a concrete didactic approach, the WFUMB identifies core characteristics which should be fulfilled [70]. Teaching methods should be easily accessible to as many students as possible so that they are perceived by many learners. In addition, the structured concept should be communicated transparently to the students. For the benefit of uniform ultrasound training and less discrepant competence levels among graduating students, the approach used should be as standardizable and reproducible as possible [70]. This also facilitates the implementation of successful training programs for other institutions. In the study of students’ learning behavior, an influence of the learning type on the learning success of students could be concluded [8]. Gender, on the other hand, showed no influence on the choice of preferred teaching method or learning success under different approaches [82]. However, in order to appeal to as many learning types as possible and thus achieve the greatest possible learning effect for all students despite their individuality, different didactic methods should ideally be used in combination [83].

### Traditional learning in medical education

Traditional medical education has historically relied on structured didactic lectures, textbooks, and instructor-led demonstrations. These pedagogical methods provide a standardized knowledge transfer mechanism, ensuring that students acquire a uniform foundation in medical sciences. However, evidence suggests that passive learning methods, such as lectures, fail to promote active engagement, critical thinking, and skill retention necessary for clinical proficiency [70]. Ultrasound education, in particular, necessitates a combination of theoretical knowledge and hands-on practice, where traditional methods often fall short in fostering psychomotor skills. Consequently, the shift towards integrative learning methodologies such as problem-based learning (PBL) and self-directed study has gained traction, aiming to bridge the gap between knowledge acquisition and clinical application [84].

### Emerging technologies in medical education

Technological advancements in medical education have led to the integration of immersive learning environments, enhancing cognitive and motor skill acquisition. Virtual Reality (VR) and Augmented Reality (AR) create simulated patient encounters that allow trainees to perform ultrasound examinations with real-time feedback, improving spatial orientation and image interpretation accuracy [85]. Serious games and gamification strategies, incorporating competitive and scenario-based learning, have been shown to increase intrinsic motivation and engagement among learners [86]. Additionally, artificial intelligence (AI)-driven educational platforms dynamically adapt to individual learning trajectories by analyzing user performance, thereby optimizing instructional content to reinforce weak areas [87]. These advancements offer promising solutions to overcome the inherent challenges of ultrasound education, particularly in ensuring consistency and standardization in skill acquisition.

### The role of collaboration in medical education

Collaborative learning plays a critical role in medical training, enhancing both knowledge retention and practical application through peer interaction. Peer-assisted learning (PAL) has been demonstrated to be an effective model in ultrasound education, as students benefit from a low teacher-to-learner ratio, increased accessibility to hands-on practice, and improved confidence levels [88]. Social cognitive theories suggest that peer educators possess an increased understanding of the cognitive and emotional barriers faced by their fellow students, leading to better-targeted instructional strategies [89]. Team-based learning (TBL) and interdisciplinary collaboration further augment the educational process by simulating real-world clinical settings, fostering interprofessional communication and collective decision-making [90]. Emerging evidence underscores the role of digital collaborative platforms in medical education, which facilitate asynchronous peer discussions, case-based learning, and knowledge sharing across disciplines, thus improving competency in ultrasound diagnostics.

### Implications of blended learning, simulation, and gamification

Blended learning models, which integrate online educational resources with in-person instruction, have been widely studied in ultrasound education. Meta-analyses indicate that blended learning enhances long-term knowledge retention and psychomotor skill acquisition more effectively than either traditional lectures or e-learning alone [91]. This pedagogical model offers flexibility, allowing learners to assimilate foundational theoretical concepts asynchronously while maximizing hands-on training during practical sessions.

Simulation-based ultrasound training has emerged as a critical component in competency-based medical education. High-fidelity ultrasound simulators replicate anatomical structures and pathophysiological conditions, enabling trainees to refine their scanning techniques and diagnostic skills in a controlled environment [92]. Studies suggest that simulation training significantly reduces cognitive load during live patient encounters, enhancing procedural confidence and diagnostic accuracy [93]. However, while simulation provides an essential scaffold for early skill development, it cannot entirely replace direct patient interactions, necessitating a structured transition from simulated practice to real-world clinical applications.

Gamification in ultrasound education has gained traction as an innovative strategy to enhance engagement and learning outcomes. By incorporating elements of competition, scoring systems, and scenario-based challenges, gamification encourages active participation and knowledge reinforcement. The use of ultrasound competitions, such as the Sono Slam, has demonstrated increased student motivation, improved teamwork, and enhanced diagnostic proficiency under time-constrained conditions [94]. Despite these advantages, gamification must be carefully implemented to ensure inclusivity, as competitive environments may not equally benefit all learners. Research is needed to determine optimal frameworks that balance engagement with equitable learning opportunities.

### Limitations

Our review has several limitations that readers should consider. First, we employed a qualitative synthesis of diverse educational studies and expert opinion; such narrative analysis is inherently subjective. Our interpretations may reflect the authors’ perspectives and experiences. Second, the studies reviewed are heterogeneous in design (ranging from small cohort studies to curricula reports) and context (different institutions, healthcare systems, and student populations). This heterogeneity limits the ability to generalize findings or perform quantitative comparisons. Third, we limited our search to English-language publications, which may have excluded relevant work in other languages. Finally, this was not a fully systematic meta-analysis – while we used systematic search methods, we did not perform quantitative data pooling. Accordingly, some selection and reporting biases may remain. These factors mean that our conclusions should be applied with caution and in context; future systematic or empirical studies are needed to validate these insights.

### Future work

The future of ultrasound education must embrace technological advancements, innovative pedagogical strategies, and scalable implementation models. The integration of Augmented Reality (AR) and Artificial Intelligence (AI) in ultrasound training presents promising opportunities for enhancing real-time procedural guidance and personalized learning experiences. AI-driven educational platforms can analyze student performance, providing adaptive feedback and individualized learning paths, thereby optimizing knowledge retention and skill acquisition.

Additionally, the role of gamification in skill retention requires further exploration. While gamified learning environments have been shown to enhance engagement and motivation, their long-term impact on clinical proficiency remains uncertain. Research should focus on evaluating whether competitive learning approaches translate into sustained diagnostic accuracy and confidence in real-world scenarios. Similarly, the scalability and standardization of blended learning approaches necessitate further investigation. With medical education institutions varying widely in resources and infrastructure, studies should assess the adaptability and cost-effectiveness of blended learning models across different learning environments.

Interdisciplinary training and peer-assisted learning models represent another critical area of research. Collaborative learning between medical students, radiologists, emergency physicians, and sonographers may foster a more integrated understanding of ultrasound diagnostics and decision-making processes. Peer-led training initiatives should also be examined to determine their efficacy in improving knowledge transfer, skill development, and student confidence in ultrasound applications.

Furthermore, the fidelity of simulation-based training and its impact on real-world clinical performance warrants deeper investigation. While high-fidelity simulators provide an effective platform for skill acquisition, their direct correlation with diagnostic accuracy and procedural competency in clinical settings remains unclear. Studies should evaluate whether the increased costs associated with advanced simulation technologies are justified by improved patient outcomes and skill proficiency.

By addressing these research priorities, ultrasound education can continue to evolve, ensuring that future clinicians are equipped with the theoretical knowledge, technical expertise, and clinical reasoning skills necessary to meet the ever-changing demands of modern healthcare.

## Conclusion

This manuscript provides a comprehensive overview of the different approaches to theoretical ultrasound education in medical studies, highlighting potential benefits and limitations of traditional and innovative approaches. Diverse teaching methods cater to various learning styles and incorporate the latest technological advancements, offering flexibility and accessibility to learners. An integrated implementation in the medical curriculum not only enhances the quality of ultrasound training but also prepares students for the complex demands of modern medical diagnostics. However, the landscape of ultrasound education faces challenges such as maintaining consistency in the quality of e-learning resources, the high costs associated with advanced simulation equipsment, limited real-life patient interaction in simulations, and potential overreliance on technology. In conclusion, ultrasound education in medical studies is evolving with technological advancements and a variety of teaching methodologies. To prepare medical students effectively for modern healthcare, it is crucial to maintain a balanced approach. This approach should leverage the strengths, address the weaknesses, seize the opportunities, and be mindful of the potential threats. The ultimate objective is to equips medical students with a comprehensive blend of theoretical knowledge and practical skills.

## Supplementary Information

Supplementary Material 1.

## Data Availability

Data available within the article or its supplementary materials.
