# Supplementary material for: Contemporary perspectives and debates in student ultrasound education: exploring didactic elements and theoretical aspects
Source: BMC Med Educ. 2026 Jun 19;26:1023. doi: 10.1186/s12909-026-09089-8 (PMC13289489; doi:10.1186/s12909-026-09089-8)
Supplement: Supplementary file 1 — Supplementary Material 1. [file 12909_2026_9089_MOESM1_ESM.docx]

References – Final Included Studies in the Review

1. Heinzow HS et al. (2013). Teaching ultrasound in a curricular course according to certified EFSUMB standards during undergraduate medical education: a prospective study. BMC Medical Education, 13:84.
2. Knobe M et al. (2010). [Undergraduate curricular training in musculoskeletal ultrasound: the impact of preexisting anatomic knowledge] (in German). Zeitschrift für Orthopädie und Unfallchirurgie, 148:685–690.
3. Cremerius C et al. (2021). Team-based learning for teaching musculoskeletal ultrasound skills: a prospective randomised trial. European Journal of Trauma and Emergency Surgery, 47:1189–1199.
4. Syperda VA et al. (2008). Ultrasonography in preclinical education: a pilot study. Journal of the American Osteopathic Association, 108:601–605.
5. Knudsen L et al. (2018). Hands-on or no hands-on training in ultrasound imaging: a randomized trial to evaluate learning outcomes and speed of recall of topographic anatomy. Anatomical Sciences Education, 11:575–591.
6. Vandenbossche V et al. (2024). Anatomical knowledge enhancement through echocardiography and videos, with a spotlight on cognitive load, self‐efficacy, and motivation. Anatomical Sciences Education, 17:806–817.
7. Vandenbossche V et al. (2023). Ultrasound versus videos: a comparative study on the effectiveness of musculoskeletal anatomy education and student cognition. Anatomical Sciences Education, 16:1089–1101.
8. Miller C et al. (2022). Near-peer POCUS education evaluation. POCUS Journal, 7:166–170.
9. Hari R et al. (2024). Describing ultrasound skills teaching by near-peer and faculty tutors using cognitive apprenticeship. Teaching and Learning in Medicine, 36:33–42.
10. Yan L et al. (2022). Sonoist: an innovative peer ultrasound learning initiative on Canadian teaching hospital wards. McGill Journal of Medicine, 20.
11. Knobe M et al. (2010). Peer teaching: a randomized controlled trial using student-teachers to teach musculoskeletal ultrasound. Medical Education, 44:148–155.
12. Li JJ et al. (2022). Comparing the effectiveness and image quality of musculoskeletal ultrasound of first-year medical students after training by student tutors versus ultrasound instructors: a pilot study. Cureus, 14:e26890.
13. Celebi N et al. (2019). Development and implementation of a comprehensive ultrasound curriculum for undergraduate medical students – a feasibility study. BMC Medical Education, 19:170.
14. Weimer JM et al. (2023). Long-term effectiveness and sustainability of integrating peer-assisted ultrasound courses into medical school – a prospective study. Tomography, 9:1315–1328.
15. Nourkami-Tutdibi N et al. (2020). Long-term knowledge retention after peer-assisted abdominal ultrasound teaching: is PAL a successful model for achieving knowledge retention? Ultraschall in der Medizin – European Journal of Ultrasound, 41:36–43.
16. Ben-Sasson A et al. (2019). Peer-teaching cardiac ultrasound among medical students: a real option. PLoS One, 14:e0212794.
17. Rong K et al. (2022). Effectiveness of near-peer versus faculty point-of-care ultrasound instruction to third-year medical students. POCUS Journal, 7:239–244.
18. Bahner DP et al. (2012). The ultrasound challenge: a novel approach to medical student ultrasound education.Journal of Ultrasound in Medicine, 31:2013–2016.
19. Boulger C et al. (2019). A national point-of-care ultrasound competition for medical students. Journal of Ultrasound in Medicine, 38:253–258.
20. Hennekes M et al. (2021). The PEGASUS games: Physical Exam, Gross Anatomy, phySiology and UltraSound games for preclinical medical education. POCUS Journal, 6:22–28.
21. Chenkin J et al. (2008). Procedures can be learned on the Web: a randomized study of ultrasound-guided vascular access training. Academic Emergency Medicine, 15:949–954.
22. Coiffier B et al. (2020). Introducing point-of-care ultrasound through a structured multifaceted module in the undergraduate medical curriculum (University of Hong Kong). Ultrasound, 28:38–46.
23. Rosenfeldt Nielsen M et al. (2021). Clinical ultrasound education for medical students: virtual reality versus e-learning, a randomized controlled pilot trial. Ultrasound Quarterly, 37:292–296.
24. Lien WC et al. (2023). The effect of e-learning on point-of-care ultrasound education in novices. Medical Education Online, 28:2152522.
25. Lin-Martore M et al. (2021). Evaluating a web-based point-of-care ultrasound curriculum for the diagnosis of intussusception. AEM Education and Training, 5:e10526.
26. Situ-LaCasse E et al. (2021). Can ultrasound novices develop image acquisition skills after reviewing online ultrasound modules? BMC Medical Education, 21:175.
27. Duarte ML et al. (2022). Comparison of ultrasonography learning between distance teaching and traditional methodology: an educational systematic review. São Paulo Medical Journal, 140:806–817.
28. Hempel D et al. (2016). Influence of case-based e-learning on students’ performance in point-of-care ultrasound courses: a randomized trial. European Journal of Emergency Medicine, 23:298–304.
29. Perice L et al. (2022). Implementation of a novel digital ultrasound education tool into an emergency medicine rotation: “UltrasoundBox”. AEM Education and Training, 6:e10765.
30. Cawthorn TR et al. (2014). Development and evaluation of methodologies for teaching focused cardiac ultrasound skills to medical students. Journal of the American Society of Echocardiography, 27:302–309.
31. Back SJ et al. (2016). Ultrasound tutorials in under 10 minutes: experience and results. AJR American Journal of Roentgenology, 207:653–660.
32. Andersen NL et al. (2023). Immersive virtual reality in basic point-of-care ultrasound training: a randomized controlled trial. Ultrasound in Medicine & Biology, 49:178–185.
33. Canty DJ et al. (2015). Ultrasound simulator-assisted teaching of cardiac anatomy to preclinical students: a pilot randomized trial of a three-hour learning exposure. Anatomical Sciences Education, 8:21–30.
34. Weimer JM et al. (2024). Insights into modern undergraduate ultrasound education: prospective comparison of digital and analog teaching resources in a flipped classroom (The DIvAN study). Ultrasound International Open, 10: (in press).
35. Kefala-Karli P et al. (2021). Introduction of ultrasound-based living anatomy into the medical curriculum: a survey of medical students’ perceptions. Ultrasound Journal, 13:47.
36. Weimer J et al. (2023). Undergraduate ultrasound training: prospective comparison of two different peer-assisted course models on national standards. BMC Medical Education, 23:513.
37. Zervides C et al. (2020). Importance of ultrasound education in the undergraduate medical curriculum: a survey of first-year students’ perceptions. Ultrasound Quarterly, 36:328–332.
38. Oteri V et al. (2020). Integration of ultrasound in medical school: effects on physical examination skills of undergraduates. Medical Science Educator, 30:417–427.
39. Tolsgaard MG et al. (2015). The effect of dyad versus individual simulation-based ultrasound training on skills transfer. Medical Education, 49:286–295.
40. Windrim C & Higgins MF (2022). Trans-vaginal ultrasound simulation: an exploratory qualitative study of learners’ perceptions. European Journal of Obstetrics & Gynecology and Reproductive Biology, 270:201–205.
41. Gradl-Dietsch G et al. (2018). Basic echocardiography for undergraduate students: a comparison of different peer-teaching approaches. European Journal of Trauma and Emergency Surgery, 44:143–152.x
